# The *mito*-QC Reporter for Quantitative Mitophagy Assessment in Primary Retinal Ganglion Cells and Experimental Glaucoma Models

**DOI:** 10.3390/ijms21051882

**Published:** 2020-03-10

**Authors:** Ines Rosignol, Beatriz Villarejo-Zori, Petra Teresak, Elena Sierra-Filardi, Xandra Pereiro, Natalia Rodríguez-Muela, Elena Vecino, Helena L. A. Vieira, Katharina Bell, Patricia Boya

**Affiliations:** 1 Department of Cellular and Molecular Biology, Centro de Investigaciones Biológicas Margarita Salas, CSIC, 28040 Madrid, Spain; 2 Experimental-Ophthalmo-Biology Group, Universidad del País Vasco, 48950 Leioa, Spain; 3 UCIBIO, REQUIMTE, Departamento de Química, Faculdade de Ciências e Tecnologia, Universidade NOVA de Lisboa, 2829-516 Caparica, Portugal; 4 CEDOC, NOVA Medical School/Faculdade de Ciências Médicas, Universidade Nova de Lisboa, 1150-082 Lisbon, Portugal

**Keywords:** autophagy, retinal ganglion cells, cell death, primary neuronal cell culture, mitophagy, *mito*-QC reporter, glaucoma

## Abstract

Mitochondrial damage plays a prominent role in glaucoma. The only way cells can degrade whole mitochondria is via autophagy, in a process called mitophagy. Thus, studying mitophagy in the context of glaucoma is essential to understand the disease. Up to date limited tools are available for analyzing mitophagy in vivo. We have taken advantage of the *mito*-QC reporter, a recently generated mouse model that allows an accurate mitophagy assessment to fill this gap. We used primary RGCs and retinal explants derived from *mito*-QC mice to quantify mitophagy activation in vitro and ex vivo. We also analyzed mitophagy in retinal ganglion cells (RGCs), in vivo, using different mitophagy inducers, as well as after optic nerve crush (ONC) in mice, a commonly used surgical procedure to model glaucoma. Using *mito*-QC reporter we quantified mitophagy induced by several known inducers in primary RGCs in vitro, ex vivo and in vivo. We also found that RGCs were rescued from some glaucoma relevant stress factors by incubation with the iron chelator deferiprone (DFP). Thus, the *mito*-QC reporter-based model is a valuable tool for accurately analyzing mitophagy in the context of glaucoma.

## 1. Introduction

The term glaucoma describes a group of etiological different diseases, that display optic nerve damage caused by the gradual loss of RGCs. This result in progressive loss of visual field and can finally lead to blindness [1]. Approximately 111.8 million people worldwide will be suffering from glaucoma by the year 2040 [2]. The main risk factors for glaucoma are an elevated intraocular pressure (IOP) and aging, however other factors such as genetics, vascular dysregulation and an autoimmune component play a role. The mechanisms of RGC death in glaucoma are still not completely understood, and different processes are under discussion [3].

Genetic studies have revealed a significant role for the autophagy process in glaucoma [4]. Autophagy is a catabolic pathway that promotes the degradation and recycling of intracellular components [5]. Thereby, proteins, lipids and whole organelles are delivered to lysosomes for degradation. Macroautophagy (further referred to as autophagy) is the best described form of autophagy, in which cytoplasmic material is engulfed in a double membrane structure, which ultimately closes to form the autophagosome. During this process, the autophagosome fuses with lysosomes allowing the degradation and recycling of cytosolic components including organelles, such as mitochondria [5].

Basal levels of autophagy are present in all cell types and are required to sustain cellular homeostasis. In response to various stressors such as metabolic stress or nutrient starvation, autophagy mediates the recycling of cell components. These are used to generate nutrients and building blocks required to sustain cell survival. Additionally, autophagy plays an important role in cellular quality control [6]. This is particularly important in post-mitotic neurons, as altered proteins and damaged organelles cannot be reduced by redistribution to daughter cells through division [7]. The deregulation of this important pathway is implicated in many diseases, ranging from cancer to immune diseases and neurodegenerative diseases [8].

Not only impaired autophagy, but also mitochondrial dysfunction, promotes cellular damage by increasing reactive oxygen species (ROS) generation or mtDNA variants. These factors have been associated with glaucoma. Likewise, mitochondrial dysfunction can be detected before RGC death occurs in glaucoma animal models [9,10,11]. Damaged mitochondria are eliminated and recycled via mitophagy, which is an important quality control mechanism in post-mitotic cells such as neurons [6,12,13]. Despite being such a relevant process, analyzing mitophagy in vivo, or even ex vivo or in vitro is still a challenging task and has been lagging behind other fields due to a paucity of robust tools. Recently, a new tool has been established to analyze mitophagy in vivo, the *mito*-QC reporter (mito-quality control). This transgenic mouse model expresses a reporter allowing the visualization of mitochondrial delivery into lysosomes during mitophagy [14,15]. The *mito*-QC reporter is a construct expressing a mCherry-GFP tag attached to the outer mitochondrial membrane FIS1 (residues 101–152) and can also be introduced into cell lines. Under normal conditions mitochondria fluoresce red and green. Upon mitophagy, mitochondria are delivered to lysosomes from autophagosomes, where solely the GFP signal is quenched by the low lysosomal pH, resulting in only red fluorescence. We have shown that this model is a very robust and useful tool to analyze mitophagy levels in the eye under physiological conditions [16].

The importance of autophagy for RGC survival in an optic nerve transection model [17,18], as well as the requirement of mitophagy for RGC differentiation [19,20] was demonstrated through our previous data. Considering that autophagy, as well as mitochondrial damage play a crucial role in glaucoma, the analysis of mitophagy in retinal ganglion cells in vitro and in vivo is of immense importance to further understand this disease. Here we use the *mito*-QC reporter for assessing mitophagy in primary retinal ganglion cells in vitro, in retinas cultured ex vivo, and in mouse models of glaucoma. Our data show that glaucoma relevant stress factors increase mitophagy in primary RGCs. Addition incubation with DFP showed an increase in mitophagy events and also lead to decreased RGC apoptosis.

## 2. Results

### 2.1. Optimization of Primary Retinal Ganglion Cell Culture Conditions

**Figure 1:**
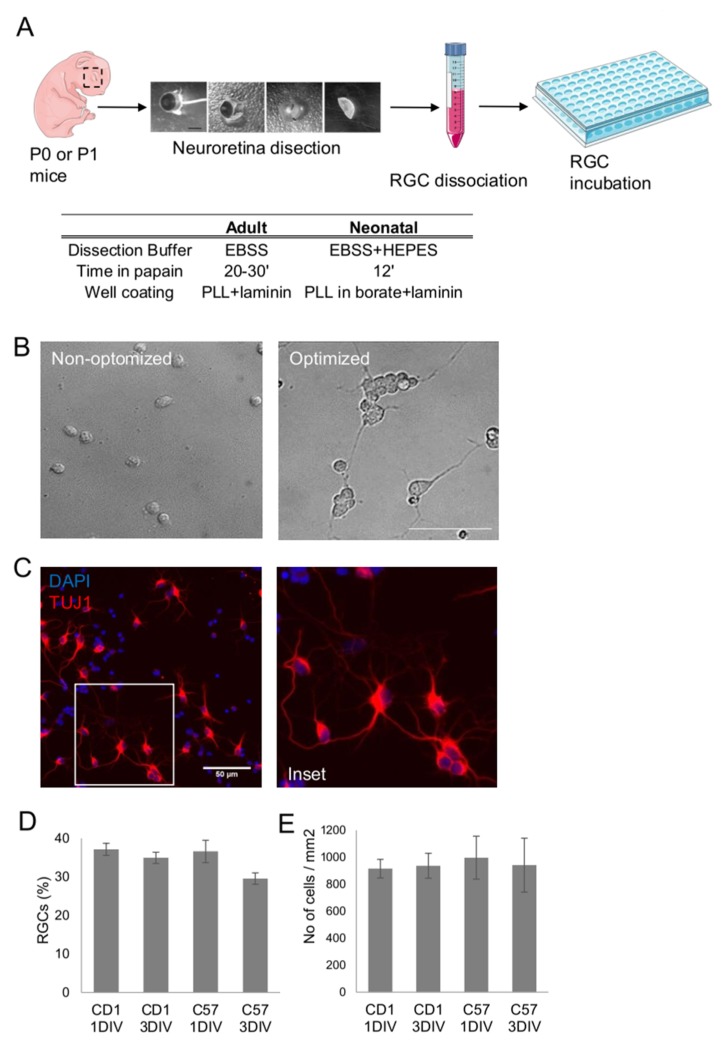
RGC culture optimization. (**A**) Experimental design and optimization steps for the improved isolation and culture. (**B**) Representative bright field images of the RGCs before and after the optimization protocol, scale bar 25 µm. (**C**) Representative β-III tubulin staining in red of RGCs 3 DIV, nuclei stained with DAPI is shown in blue, scale bar 50 µm. (**D**,**E**) % of RGCs and number of cells/mm^2^ in the primary cultures isolated from CD1 and C57 mouse strains.

We adapted a protocol from primary adult rat RGC isolation [21] and optimized the procedure to improve the survival of neonatal mouse RGCs in culture, based on 3 different steps, Figure 1A. Dissection buffer, the well coating procedure and the duration of the retinal papain dissociation were adapted. RGC morphology before and after procedure optimization is displayed in Figure 1B. β-III tubulin staining shows healthy RGCs after optimizing the protocol, Figure 1C. Doing so, we could maintain a stable culture containing almost 40% RGCs for at least 3 days in vitro (DIV), despite using a fairly basic cell culture medium (see materials and methods), Figure 1D. Moreover, RGC isolation protocol and RGC yield were validated in two different mouse strains, CD1 (an outbred strain albino) as well as C57 (an inbred and pigmented strain). Likewise, the total number of cells after one and three DIV were similar in both strains, Figure 1D. In conclusion, our optimized protocol allows the isolation and culture of primary RGCs isolated from perinatal mice from different mouse strains.

### 2.2. Susceptibility of RGCs to Glaucoma-Relevant Stressors

**Figure 2:**
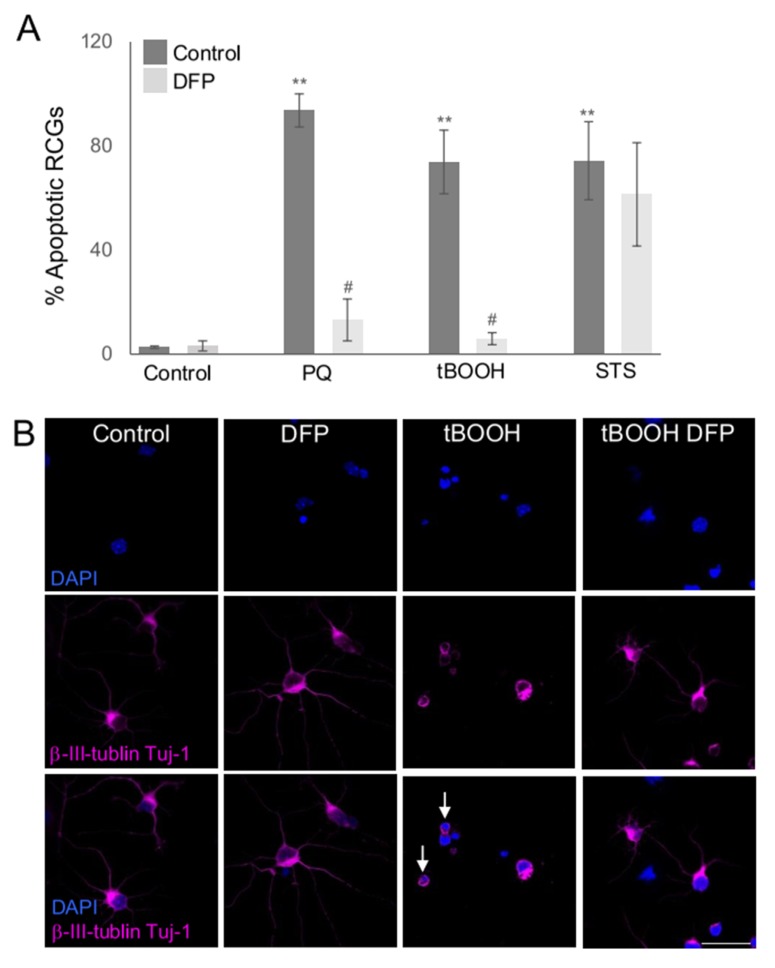
DFP rescues RGC death from oxidative stress inducers. (**A**) Primary RGCs were incubated with 2 mM PQ, 200 µM tBOOH and 3.5 µM STS, in the presence or absence of 0.25 mM DFP for 24 h. After the incubation cells were fixed and stained with β-III-tubulin (TUJ1) and DAPI. The % of apoptotic RGCs was determined by counting TUJ1 positive cells with condensed apoptotic nuclei. The number of RGCs was counted in at least 5 fields of three different experiments. ** *p* < 0.01 vs. control; # *p* < 0.05 vs. treatment. (**B**) Representative images of cells treated as in A stained with β -III-tubulin (TUJ1, magenta) and nuclei (DAPI in blue). Examples of apoptotic nuclei are depicted with white arrows. Scale bar 50 µm.

To validate the model, we used the improved primary culture to explore the susceptibility of RGC to different glaucoma relevant stressors. Oxidative stress inducers paraquat (PQ) and *tert*-butyl hydroperoxide (tBOOH), an organic peroxide generating ROS, were used to promote cell death [22], and staurosporine (STS), a general kinase inhibitor, as apoptosis inducer. We found that all the applied stress factors lead to a significant increase in primary RGC apoptosis, assessed by nuclear condensation with DAPI, after 24 h of incubation, Figure 2A. More interestingly, we found that the iron chelator deferiprone (DFP), which is a molecule often used to induce mitophagy [15,23], rescued RGC population following PQ and tBOOH treatment, Figure 2A. It however failed to rescue RGCs from damage induced by STS, Figure 2A, showing that the cytoprotective effects are stimulus-specific.

### 2.3. The mito-QC Reporter as a New Tool for Mitophagy Assessment In Vitro

Because DFP significantly protected RGC against cell death, we wanted to investigate how mitophagy was modulated taking advantage of the *mito*-QC reporter, a recently described tool for an accurate mitophagy assessment. Mitophagy is quantified by assessing the number of red-only puncta, representing the mitolysosomes [15].

**Figure 3:**
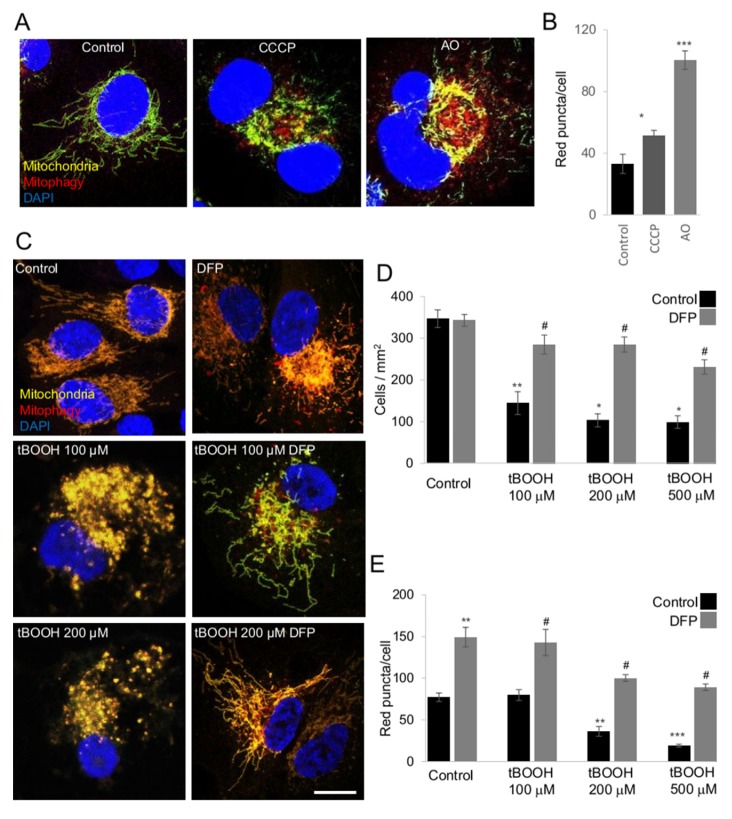
Mitophagy monitoring with *mito*-QC reporter cells. ARPE19 *mito*-QC cells were incubated with different mitophagy inducers, 10 µM CCCP, antimycin 10 µM plus oligomycin 1 µM for 24 h. Representative confocal images of ARPE19 *mito*-QC cells after 24 h of incubation with the indicated treatments, as well as corresponding control cells are displayed in (**A**) scale bar 10 µm. (**B**) Quantification of red-only puncta in cells treated as in A. (**C**) Representative confocal images of control cells, cells incubated with 0.25 mM DFP, cells treated with the indicated doses of tBOOH with or without the presence 0.25 mM DFP for 24 h, scale bar 10 µm. (**D**) Quantification of the number of viable *mito*-QC ARPE-19 cells treated as in C. (**E**) Quantification of the number of puncta (red-only dots per cell) in cells treated as in C. * *p* < 0.05 vs. control, ** *p* < 0.01 vs. control; *** *p* < 0.001 # *p* < 0.05 vs. treatment.

Before examining mitophagy in primary RGCs, we at first analyzed and validated the reporter system using a cell line stably expressing the *mito*-QC reporter. Therefore, we first assessed how several already described mitophagy inducers modulated mitophagy in *mito*-QC reporter ARPE-19 cells. As expected, the mitophagy inducers carbonyl cyanide *m*-chlorophenyl hydrazone (CCCP), antimycin/oligomycin (AO) and deferoxamine (DFO) induced a significant increase in red puncta in the reporter cells, showing the stability and the reproducibility of the *mito*-QC reporter for our purposes (Figure 3A,B).

Since incubation with DFP rescued RGC viability from treatment with glaucoma related stress factors, we used the *mito*-QC cells to quantify mitophagy and assess its protective role in greater detail using the *mito*-QC ARPE-19 cells. tBOOH treatment greatly reduced cell viability, which was restored by the presence of DFP, Figure 3C,D and Figure S1. More importantly, DFP increased the number of red-only puncta, indicating that it is inducing mitophagy, Figure 3E. This data indicates that the *mito*-QC reporter, as proposed, nicely indicates increases in mitophagy and that mitophagy activation can function as cytoprotective mechanism under stress conditions.

### 2.4. The mito-QC Reporter as a New Tool for Mitophagy Assessment in Primary RGCs

**Figure 4:**
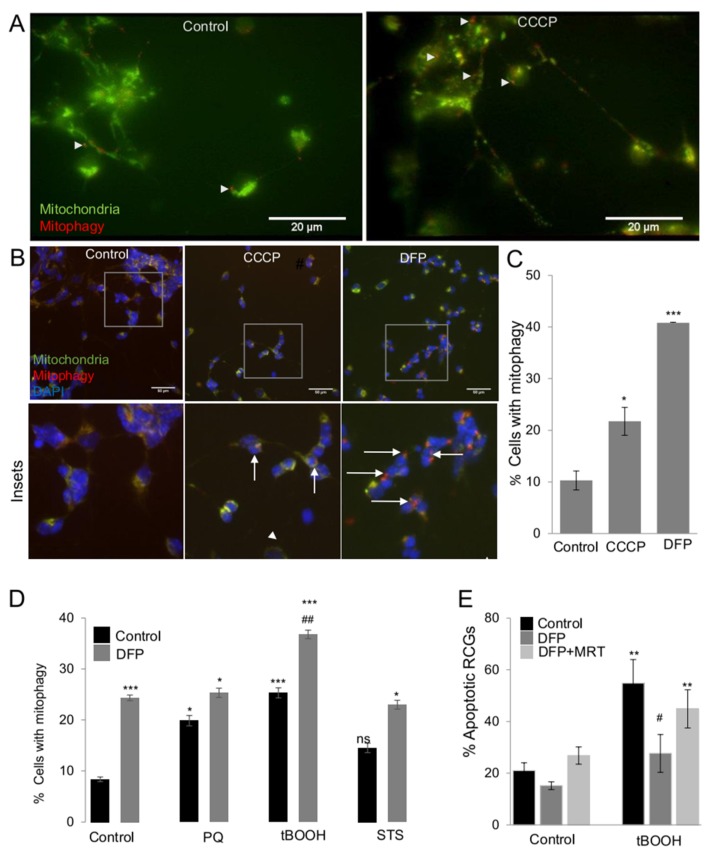
Mitophagy induction and rescue from oxidative stress in *mito*-QC primary RGCs. (**A**) Primary RGCs isolated from P0 *mito*-QC mice were incubated with CCCP at 10 µM for 3 h. Representative images in live RGCs, note the red-only puncta that are labeled with white arrows. Scale bar 20 µm. B. Primary RGCs isolated from P0 *mito*-QC mice were incubated for 24 h with 25 µM CCCP and 2.5 mM DFP. Representative epifluorescence images are shown in (**B**), where the red puncta indicative of mitophagy are depicted with white arrowheads. The quantification of the % of mitophagy positive cells is depicted in (**C**). (**D**) Primary RGCs isolated from P0 mice were treated for 24 h with 2 mM PQ, 200 µM tBOOH and 3.5 µM STS, in the presence or absence of 0.25 mM DFP for 24 h. After the incubation cells were fixed and stained with b-III-tubulin (TUJ1) and DAPI. The % of apoptotic RGCs was determined by counting TUJ1 positive cells with condensed apoptotic nuclei. The number of RGCs was counted in at least 8 fields of three different experiments. * *p* < 0.05 vs. control; *** *p* < 0.001 vs. control, # *p* < 0.05 vs. treatment, ## *p* < 0.01 vs. treatment. (**E**) Primary RGCs isolated from P0 *mito*-QC mice were incubated with 200 µM tBOOH in the presence or absence of 0.25 mM DFP and 1 µM MRT for 24 h. After the incubation cells were fixed and stained with b-III-tubulin (TUJ1) and DAPI. The % of apoptotic RGCs was determined by counting TUJ1 positive cells with condensed apoptotic nuclei. The number of RGCs was counted in triplicates in 4 fields per condition (>200 RGCs per condition) * *p* < 0.05 vs. control; ** *p* < 0.01 vs. control # *p* < 0.05 vs. treatment.

Subsequently, primary RGCs derived from *mito*-QC animals were used as a tool for analyzing mitophagy and mitophagy induction as cell protective mechanism in a more physiologically relevant model for glaucoma. In the past, mitophagy assessment in primary neurons and in RGCs has been a very cumbersome task and was only possible through indirect methods. First, we tested whether primary RGCs from the *mito*-QC animals presented red dots following CCCP treatment in live neuronal cultures by live-imaging microscopy. Increased numbers of red-only dots were displayed in RGCs in the presence of CCCP (Figure 4A), indicating mitophagy activation. Thus, RGCs from the *mito*-QC animals can be a useful tool to assess mitophagy.

Next, primary RGCs isolated from *mito*-QC reporter mice were treated with different mitophagy inducers at various concentrations. Increased numbers of RGCs displayed mitophagy puncta following mitophagy activation, Figure 4B,C. Interestingly, PQ and tBOOH also increased the % of RGCs with red dots while STS did not, Figure 4D. Co-incubating with DFP further increased mitophagy for all the treatments. This data therefore suggests that mitophagy could be increased as a pro-survival mechanism in RGCs challenged with oxidative stress inducers but not with a general protein kinase inhibitor, Figure 4D. In support of this hypothesis the pro-survival effect of DFP against tBOOH is completely abolished in the presence of the Ulk1 inhibitor MRT68921, indicating that functional autophagy is needed for DFP to protect RGCs from tBOOH, Figure 4E. In conclusion, primary cultures derived from *mito*-QC retinae are a useful tool to analyze mitophagy in primary RGCs in vitro. Likewise, it was demonstrated in an accurate manner that CCCP and DFP promote mitophagy in primary RGCs and that DFP rescued primary RGCs from oxidative challenge.

### 2.5. Ex vivo Explants from mito-QC Reporter Mice as Model System for Mitophagy Assessment

**Figure 5:**
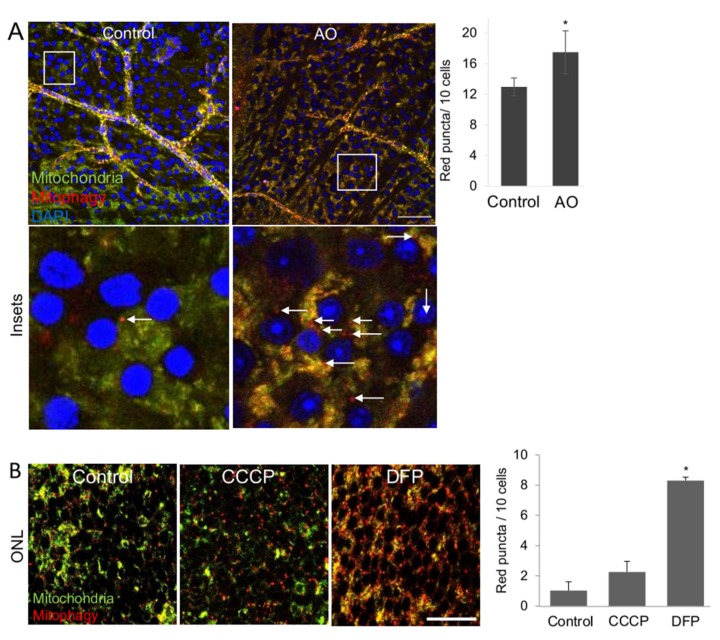
Ex vivo retinal explants as tool for mitophagy assessment. (**A**) Retinal explant cultures treated with antimycin 10 µM plus oligomycin 1 µM for 8 h. Graph shows the number of red-only puncta per 10 nuclei in the RGCL, scale bar 25 µm. (**B**) Retinal explants culture with 10 µM CCCP and 1mM DFO for 8 h. Graphs show the number of red-only puncta per 10 nuclei in the ONL, scale bar 10 µm. ONL, Outer nuclear layer; RGCL, retinal ganglion cell layer. * *p* < 0.05.

Next, we determined mitophagy in RGCs in ex vivo explant retinal cultures, a key tool frequently used to perform neuroprotection studies targeting RGCs. Explants incubated with the mitophagy inducer AO showed more red-only puncta in the RGC layer (RGCL) in comparison to the control retinae in culture (Figure 5A).

Not only the RGC layer, but also other retinal layers such as the outer nuclear layer (ONL) demonstrated increased red-only puncta. Treatment with CCCP and DFP resulted in increased red puncta in the ONL, Figure 5B, which indicates that ex vivo retinal cultures can be used for mitophagy analysis throughout the whole retina. We counted lower levels of basal as well as induced mitophagy per cell in the ONL in comparison to the RGC layer. It is however not surprising to find different amounts of basal as well as induced mitophagy different cell types of one tissue. It has been well demonstrated that mitophagy levels do not only differ depending on the tissue, but also can be highly variable within different cell types of one tissue [24].

### 2.6. CORM-A1 Induces Mitophagy In Vitro, Ex Vivo and In Vivo

**Figure 6:**
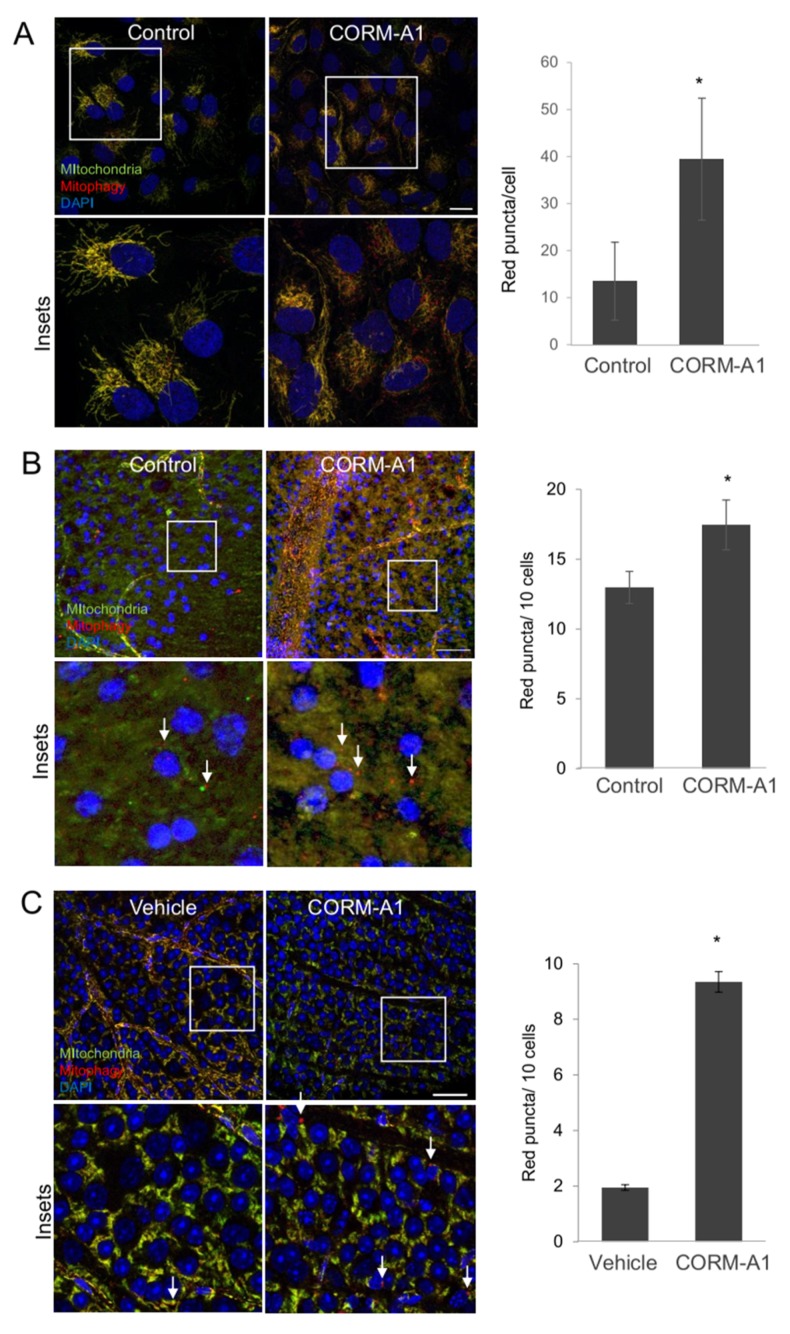
CORM-A1 induces mitophagy, in vitro, ex vivo and in vivo. (**A**) ARPE-19 *mito*-QC cells were incubated with CORM-A1 for 24 h and the number of red-only puncta was quantified in at least 30 cells from the confocal images. Scale bar 20 µm. (**B**) Retinal explants from the *mito*-QC animals were incubated with CORM-A1 for 8 h. Representative confocal images at the quantification of the number of red-only dots are displayed. Magnifications show representative pictures of mitophagy positive cells in the RGCL, scale bar 25 µm. C. *mito*-QC animals were treated with daily intraperitoneal CORM-A1 injections for 7 subsequent days, before analyzing mitophagy red-only puncta in the RGC layer. Figure 6(**C**) shows pictures of the RGC layer as an overview as well as a magnification and the graph display the quantification of red-only puncta in the RGCL, labeled with white arrows. Scale bar 25 µm. RGCL, retinal ganglion cell layer. * *p* < 0.05.

Carbon monoxide (CO) is an endogenous gasotransmitter promoting cytoprotection and autophagy. Making use of the *mito*-QC reporter cells we wanted to determine whether the CO-releasing molecule CORM-A1 can induce mitophagy in RGCs. First, we analyzed the effect of CORM-A1 on *mito*-QC reporter cells. As shown in Figure 6A, CORM-A1 treatment induced an increase in the number of red-only puncta. A similar effect was observed in the retinal ganglion cell layer (RGCL) from ex vivo retinal explants derived from *mito*-QC mice incubated with CORM-A1 (Figure 6B). Likewise, there was a significant increase in mitophagy levels in the RGCL and in the ONL of explants treated with CORM-A1 in comparison to the control explants Figure 6B, and Figure S2.

Finally, we further determined the effect of CORM-A1 on RGC mitophagy in vivo. On 7 consecutive days, animals were injected intraperitoneally with CORM-A1 for 7. The red-puncta analysis showed a significant 5.2-fold increase in mitophagy in the RGCL of the animals injected with CORM-A1, Figure 6C. In conclusion, our data shows increased mitophagy in the retina after ex vivo and in vivo CORM-A1 administration.

### 2.7. Increased Mitophagy after Retinal Ganglion Cell Damage In vivo

**Figure 7:**
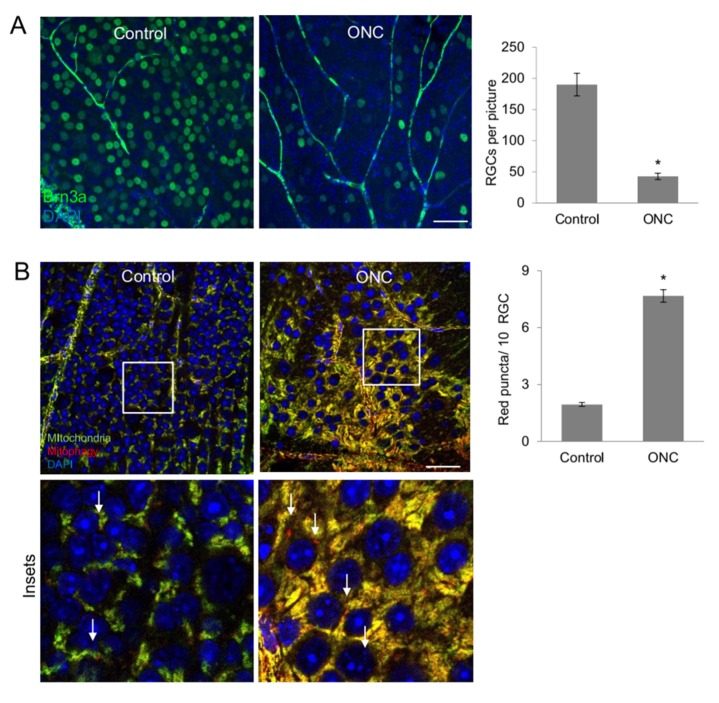
Optic nerve crush damage increases RGC mitophagy. ONC was performed in the *mito*-QC mice. 7 days after the damage, RGCs were counted in flatmounts after Brn3a (green) and DAPI (blue) staining. (**A**) Representative Brn3a staining of a control as well as an ONC retina. Significantly less RGCs were present in the retinae 7 days after ONC (* *p* < 0.05). (**B**) Mitophagy puncta in the RGC layer were assessed 7 days after the ONC. Pictures show the RGC layer in the control as well as the ONC eye, with representative magnifications. Scale bar 25 µm. The arrows point towards red mitophagy puncta.

The role of mitophagy during RGC damage and recovery is still under discussion. Therefore, we analyzed the levels of mitophagy in RGCs after optic nerve crush in the *mito*-QC animals. For this, we performed ONC (optic nerve crush) and analyzed red puncta in the RGCL 7 days after the crush. Confirming previous studies, our data shows massive decrease in Brn3a-positive RGCs 7 days after ONC, Figure 7A. More importantly, our masked analysis revealed a significant increase in mitophagy puncta in the RGCL after ONC, Figure 7B. The number of mitophagy events was 4.9-fold higher in the ONC RGCL in comparison to the uninjured eye. The RGCL is composed mainly from RGCs but also limited numbers of displaced amacrine cells or displaced astrocytes can be detected. In conclusion, the *mito*-QC reporter-based model is efficient to assess and quantify mitophagy in vivo following retinal ganglion cell damage.

## 3. Discussion

We showed that the *mito*-QC reporter is an accurate tool to quantitatively assess mitophagy in vitro in cell lines and primary RGCs. Additionally, we demonstrated that incubation of RGCs with DFP leads to mitophagy induction and can decrease RGC apoptosis under different glaucoma-related stress conditions. We also found that mitophagy levels increased in the RGCL ex vivo and in a classical model of optic nerve damage in vivo.

### 3.1. Improvement of primary RGC Culture

Isolating and culturing primary RGCs is a valuable tool for studying all diseases concerning the optic nerve, such as glaucoma. Previous studies have shown that primary RGC cultures from adult animals can preserve similar neurotrophic characteristics as found in vivo [25]. We have also tested the survival and branching capacity of these RGCs when growing in different substrates [26], and how RGC survival can be enhanced by Müller glia [21,25,27]. Here we present an optimized protocol for culturing RGCs from young mice as the previous procedure used for adult rats lead to very variable and low RGC yield when applied to neonatal RGCs. Within the same cell culture isolation there were differences in cell morphology and adherence, indicating a non-robust protocol. First, we increased RGC yield by adding borate buffer to the coating solution. This changes the pH to 8.5, which increases the interaction between the plastic-based surface of the well plate and poly-L-lysine, resulting in significantly increased and unified adherence of the retinal cells to the wells. Borate buffer is widely used as a key component for maintaining reliable and viable primary neuronal cultures [28]. Interestingly, we found that adding borate buffer also enhanced the adherence and RGC yield in glass coverslips. Adding HEPES to the isolation buffer also increased RGC viability. HEPES provides a more stable pH for dissection, improving cell viability of the more delicate young retina. Papain is typically applied for dissociation of neuronal tissues, as its damaging properties are less than other proteolytic enzymes. However, long incubation time can damage the cells in a non-reversible manner and therefore lead to cell death and tissue loss. Herein it was possible to decrease papain incubation time without decreasing the final cell yield *per* retina, thus adapting the protocol for neonatal retinas. The combination of these three optimized steps led to a retinal culture that displayed improved morphology and lower percentage of cell death, even after 3 DIV.

Other studies isolating RGCs without using an additional selection step, such as immunopanning or magnetic sorting, lead to very low yields of RGC in retinal cell cultures. A recent study using a slightly different dissociation protocol and media composition shows a yield of approximately 5% of RGCs in the mixed cell culture [29]. The papain incubation time was 30 min. Other studies aiming to improve RGC yield in mixed retinal cell cultures also incubated papain for 30 min, however, do not show the % of RGCs achieved in the whole culture, making direct comparisons difficult [30]. Using our isolation protocol and carefully handling the cells led to a viable culture that consists of about 30% RGCs. One step or two step immunopanning to purify RGCs leads to higher RGC purity, however, comes with a significantly increased cost and may not be desirable, since it limits the potential beneficial glial-RGC interactions in culture. RGC isolation in rats showed purity of up to 90% RGCs when using the immunopanning method [31], thus reducing the cell yield per retina ultimately leading to the use of more animals to get the same number of RGCs. To further improve culture conditions for longer term cultures, adding supporting factors such as BDNF or CNTF to the medium could be beneficial [25,32].

### 3.2. mito-QC Cells as Useful Tool for Studying Mitophagy in RGCs

Mitochondrial RGC damage and autophagy play an important role in glaucoma, thus more efficient tools are necessary to quantitatively assess mitophagy in this context. Our results show, that primary RGCs derived from *mito*-QC reporter mice *in vitro*, as well as ex vivo and in vivo can fill the gap of this so far unmet need. Previously, we published a flow cytometric approach to study mitophagy not only in cell lines, but also tissue, using MitoTracker Deep Red [33]. *Mito*-QC offers several advantages, not only compared to MitoTracker Deep Red, but also other models to measure mitophagy, such as mt-Keima, which partially have been summarized in some recent reviews [34,35]. First, *mito*-QC aims to provide a “real-time” analysis the mitochondria-containing autophagosome delivery to the lysosome to form the autolysosome. In comparison to the MitoTrackerDeep Red approach, which shows a decrease in staining upon mitophagy induction, using flow cytometry, this process can be neatly visualized in cells and tissues containing the *mito*-QC reporter. Tissues can be fixed and analyzed, which is not possible in the mt-Keima model giving the possibility co-staining for specific cell types to localize the mitophagy puncta in greater detail. However extreme care has to be taken to avoid pH shifts, which is a limitation of the model.

Additionally, *mito*-QC provides information on mitochondrial morphology and distribution within the analyzed tissue. With help of the *mito*-QC reporter we recently demonstrated mitophagy throughout the retina in the embryonic stages, although general autophagy is more prominent during these developmental ages [16]. More importantly, mitophagy is not only required but also essential to promote RGC differentiation in vivo, as autophagy (Atg5) and mitophagy (NIX)-deficient animals displayed reduced number of RGCs [20]. This developmentally regulated mitophagy induces a metabolic change that is essential for proper RGC differentiation. Autophagy-deficient retinas display altered neurogenesis, including ectopic mitosis and aberrant axonogenesis. Using retinal cryosections of *mito*-QC animals ee previously showed mitophagy activation in developing RGCs can be clearly observed in at the stages where RGC neurogenesis occurs [16], thus corroborating the usefulness of this tool for mitophagy assessment in vivo. With this study we have generated information with more detailed analysis of mitophagy in the RGCL in basal and stress conditions, demonstrating the robustness of this tool. Likewise, it was also possible to determine stress-induced mitophagy in the whole retina in vivo.

We validated the use of several mitophagy-inducing compounds as new tools to induce mitophagy in vivo, including CCCP, and AO. Mitophagy activation of the carbon monoxide (CO)-releasing molecule CORM-A1 was tested in different retinal layers. CO is an endogenous gasotransmitter, which promotes cytoprotection and homeostasis and limits inflammation [36]. It can administered by CO gas (inhalation or saturated solutions) or by CO-releasing molecules (CORM) [37]. CO has been described as neuroprotective for RGCs [38,39,40]. Likewise, CO is known to induce autophagy [41,42], as well as mitophagy and mitochondrial biogenesis [22,43], indicating its role in mitochondrial function modulation. Recently studies show that CORM-A1 promotes autophagy in mammal cells and yeast [44]. Using CORM-A1 in the *mito*-QC model system, we demonstrated mitophagy induction in different layers of the retina including the RGCs, not only ex-vivo, but also in vivo.

### 3.3. RGC Damage and Mitophagy

Mitochondrial homeostasis is proven to be critical for ocular health and important in the context of glaucoma. It serves as a possible therapeutic target [45] and genetic studies reveal that impaired mitophagy plays a role in glaucoma [46]. We demonstrate an increase in mitophagy in the RGCL after ONC damage. We also show increased cell death of primary RGCs after different glaucoma relevant stressors such as the oxidative stress inducers tBOOH or PQ. Interestingly, when treating the stressed cells with the mitophagy inducer DFP, we observed RGC rescue. Oxidative stress plays an important role in glaucomatous RGC damage. Increased oxidative stress markers have been detected in glaucoma patients [47] and confirmed in a recent meta-analysis [48]. Targeting oxidative stress in glaucoma therefore is in discussion as being a valid therapeutic target option [49]. Rescue of RGC by antioxidants has been demonstrated in the past. RGC survival in an ONC model was promoted by reducing oxidative stress through Nrf2 overexpression, a main anti-oxidative gene, [50]. Using the DBA/2J glaucoma model, another study showed increased RGCs survival by inhibiting DRP1, a mitochondrial fusion protein, and therefore reducing oxidative stress [51]. Induction of mitophagy could decrease oxidative stress in different contexts [52,53]. Beneficial effects of mitophagy induction to rescue damaged RGCs has also been demonstrated. Using other glaucoma models, previously published studies have demonstrated a beneficial effect for RGC survival when increasing mitophagy in IOP related glaucoma models, either directly, e.g., by Parkin overexpression [54] or indirectly, e.g., by deletion of mitochondrial *Ucp2* [55]. Although not proven so far, it is tempting to believe that the protective effect is due to the elimination of damaged mitochondria and possibly reduction of oxidative stress. More studies are needed to support this hypothesis.

Further research is necessary to overcome the limitations of this study. Using not only mitophagy activators, but also other “selective-autophagy” activators, one has to take into account the possible bystander induction of autophagy, This has been nicely described using the Keima-fused proteins [56] and also could influence the outcome of this study. The presented work would also benefit from using selective mitophagy inhibitors to underline the fact that mitophagy induction is cytoprotective against various stress factors. However, so far, the field is still lacking substances for specific mitophagy inhibitors. Measurements of reactive oxygen species should be added to future studies.

In conclusion, our study demonstrates that the *mito*-QC reporter is a very robust tool to analyze mitophagy in primary RGCs cultures, ex vivo and in vivo. Moreover, our results give hints that mitophagy induction could be cytoprotective for RGCs, however, further research is needed. Within this area, it would be very interesting to explore whether mitophagy activation can serve as cytoprotective response through promoting metabolic changes. This would be in line with the findings in embryonic RGCs, which ultimately promote RGC survival by eliminating damaged mitochondria [20]. This tool opens new research avenues for glaucoma and other neurodegenerative diseases. Boosting autophagy, or more specifically mitophagy could serve as a valuable target for certain glaucoma patient population, the literature has already shown some valuable input, but further research needs to be performed.

## 4. Materials and Methods

### 4.1. Animals and Genotyping

Animals were housed, cared and euthanized in accordance with the European guidelines and experiments were approved by the CIB ethics committee for animal experimentation and the Comunidad de Madrid, codes PROEX 232/17 (approval date 28.1.2018) and PROEX226/16 (approval date 22.9.2016). Mice were housed in the animal facility of CIB with *ad libitum* food and water in 12/12 h light dark cycle. Adult CD1 and C57/BL6 animals from both sexes were obtained from CIB animal facility. *mito*-QC animals [14] were kindly provided by Dr Ian Ganley (School of Life Sciences, The University of Dundee, Dundee, Scotland) and in-house bred. Briefly, the mice have an introduced CAG promoter cassette with the open reading frame for the mCherry-GFP-FIS1 fusion protein including a Kozak sequence (GCCACC) into the mouse Rosa26 locus in mice with a C57BL/6 background. Genotyping was performed by diagnostic end-point PCR using genomic DNA isolated from tissue biopsy specimens with the following sets of forward and reverse primers: set 1, 5′-CAAAGACCCCAACGAGAAGC-3′ and 5′-CCCAAGGCAC ACAAAAAACC-3′; and set 2, 5′-CTCTTCCCTCGTGATCTGCAACTCC-3′ and 5′-CATGTCTTTA ATCTACCTCGATGG-3′. 

### 4.2. Cell Lines

ARPE-19 (Human Pigment Epithelial cell line) stably expressing the *mito*-QC reporter cell lines, generated in the laboratory of Dr. Ian Ganley, as described in [15] were grown in DMEM medium: F12 (1:1) with 15% FBS, 1% glutamine (2 mM) and 1% penicillin-streptomycin (0.5 mg ml). The culture media were filtered with 0.22 μm Stericup Filters (SCGPU02RE, Millipore, Burlington, MA, USA) and the cells were kept in incubators at 37 °C with 5% CO2. Cells where cultured with 800 μg/mL Hygromycin B (10,453,982, Gibco, Carlsbad, CA, USA) to select only those cells expressing the *mito*-QC reporter.

### 4.3. Retinal Cell Isolation and Culture

#### 4.3.1. Coating of Well Plates

The previous day, the required wells of a 96 well plate (Screenstar microplate, 655,866, Greiner bio-one, Monroe, NC, USA) were coated with 10 µg of poly-L-lysine (PLL, P1399, Sigma-Aldrich, St. Louis, MO, USA) in sterile H_2_O with 50 mM borate buffer (Pierce 20× borate buffer, 28341, Pierce Biotechnology, Rockford, IL, USA) and incubated overnight at 37 °C. The next day the wells were washed three times with sterilized H_2_O and coated with 0.5 µg laminin (L2020, Sigma-Aldrich) diluted in sterile 1× PBS. The plates were incubated for at least 3 h in an incubator at 37 °C with 95% CO_2_. The laminin solution was removed directly before adding the final cell suspension, without washing.

#### 4.3.2. Isolation of Retinal Cell Culture

For RGCs isolation, P0-P1 mice were used. The eyes were isolated in cold dissection buffer made of HBSS (14,170,088, ThermoFisher) containing 2 mM HEPES (H3784, Sigma-Aldrich). Using fine tweezers (91150-20, Fine Science Tools GmbH, Heidelberg, Germany) the retina was separated first from the sclera and pigment epithelium, then from the *ora serrata* and, at last, from the lens under a binocular dissecting microscope (MZ7.5, Leica, Heidelberger, Germany). The obtained retinas were placed in the dissection buffer.

The dissociation of the retina was performed with adaptations from the Papain Dissociation kit (PDS LK003150, Worthington Biochemical Corp., Lakewood, NJ, USA). EBSS (32 mL) was added to the albumin-ovomucoid inhibitor and equilibrated in the incubator. EBSS (5 mL) was added to the vial containing papain (20 U/mL) and placed in a water bath at 37 °C for approximately 10 min. EBSS (500 µL) was added to the vial containing DNase (2000 U/mL). For each pair of eyes 20 µL of the DNase solution were added to 200 µL papain solution in a 2 mL tube. Two retinas were added per tube and placed in the incubator for 12 min. The tissue was processed by slowly pipetting the mixture up and down with a 1 mL pipette tip. Cell suspension was centrifuged at 130× *g* for 5 min at RT. After removal of the supernatant the cell pellet was resuspended in a media consisting of 170 µL EBSS and 19 µL albumin-ovomucoid inhibitor solution. Next, a density gradient centrifugation was performed by adding the cell suspension carefully on top of 200 µL of albumin-ovomucoid inhibitor solution in a new 2 mL tube. This density gradient was centrifuged at 70× *g* for 6 min. After removal of the supernatant, the cell pellet was resuspended in 2 mL of primary RGC media: Neurobasal (21,103,049, ThermoFisher), 0.5% gentamicin (15710-049, Gibco, Carlsbad, CA, USA), 0.25% L-glutamine (25030-024, Gibco) and 2% B27 (17,504,044, ThermoFisher). From this cell suspension, 10 µL were taken to perform a cell count. Cells were counted using a Neubauer counting chamber after adding 10 µL of trypan blue (15-250-061, ThermoFisher). Finally, after some optimization to achieve better survival 25.000 cells were plated into each well of the screen star plate with a final volume of 100 µL media.

#### 4.3.3. DIC Imaging and Analysis

To study the growth of neuronal cells in the culture without the need of fixing the cells, DIC pictures were acquired at 1 and 3 DIV. 5 pictures (×40 objective) were taken per well with a fluorescence microscope (AF6000 LX Widefield Multidimensional Microscopy System, Leica, Heidelberger, Germany), epifluorescence and transmitted light, consisting of an inverted Leica DMI6000B microscope and a 9100-O2 CCD (Hamamatsu Photonics K.K., Japan) and the software LAS X. Cells with neuronal projections were identified as neuronal cells.

#### 4.3.4. Immunofluorescence Staining

Neuronal marker β-III-tubulin (TUJ1, MMS-435P, Biolegend, San Diego, CA, USA) staining was performed to label RGCs as described before [57]. Briefly, cells were fixed with 4% PFA (in house production from 1,040,051,000, Merck, Darmstadt, Germany) for 15 min, permeabilised with SDS 0.1% for 30 min and incubated with the primary antibody (1:500) overnight at 4 °C in a humid chamber. The secondary antibody was incubated for 1 h at RT (1:200), followed by a DAPI solution for 10 min (1 µg/mL in 1× PBS). 10 pictures per well were taken. The ImageJ Cell Counter Plugin was used to count cell nuclei and count neuronal cells. 

### 4.4. Drugs and Treatments

Cells were incubated at the indicated times and doses, control cells where treated with vehicle, DMSO. We used paraquat (856,177, Sigma), *tert*-butyl hydroperoxide (tBuOOH, B2633, Sigma) and staurosporine (STS, S4400, Sigma), iron chelator deferiprone (DFP, 374409, Sigma), DFO (deferoxamine, D9533, Sigma), 3-chlorophenyl)-hydrazono-malononitrile (CCCP, C2759, Sigma), antimycin A (A8674, Sigma) and oligomycin A (A8674, Sigma) and the CO (carbon-monoxide) releasing molecule CORM-A1 (SML0315, Sigma), MRT68921 a Ulk1 inhibitor (5780, Tocris, Avonbridge Trading Estate, Bristol, UK). After the treatments cells were fixed with 3.7% PFA containing 200 mM HEPES and visualized with the multidimensional microscope (Leica AF6000 LX Widefield Multidimensional Microscopy System) and the SP5 microscope (Leica TSC SP5 confocal microscope). *Mito*-QC mice were intraperitoneally daily injected either with CORM-A1 (2 mg/kg body weight dissolved in PBS and applied in 200 µL end volume) or PBS (200 µL) for 7 subsequent days (n = 7 per group). The mice were sacrificed, and retinae were treated as described below.

### 4.5. Retinal Explants

*Mito*-QC animals were used to determine mitophagy in retinal ex vivo explant cultures under different treatment conditions as described. After sacrificing the animals through cervical dislocation, retinae were isolated from the eyes as described previously [58]. Retinae were placed in 24 well plates and incubated with DMEM (Dulbecco’s Modified Eagle Medium; 41966-029, Gibco) with 1% glutamine (2 mM, 25,030, Gibco), 1% penicillin-streptomycin (0.5 mg/mL, 11,568,876, Gibco) and 1 µM of insulin (I2643, Sigma) with the indicated treatments and time. After incubation flatmounts were washed with PBS and fixed in 3.7% PFA containing 200 mM HEPES for 1 h at RT and subsequently washed 3× 5 min with PBS. Flatmounts were mounted in Vectashield (H-1000-10, Vector Laboratories INC, Burlingame, CA, USA). Pictures were taken with an SP5 microscope (Leica TSC SP5 confocal microscope) with a ×63 objective.

### 4.6. Mitophagy, Red-Only Puncta Quantification

The quantification in the *mito*-QC ARPE cells and was done manually using the cell counter plugin from ImageJ to assess red only-puncta for each confocal plane. For retinas four pictures were taken per retina, red-only puncta and nuclei were counted manually in every Z stack for the RGC layer, with a ScatterPlot plugin based on a measure of colocalization (GFP-mCherry) in the ONL and the RGCL.

### 4.7. Optic Nerve Crush and Histological Flatmount Evaluation

Mice were first anaesthetized with a weight adapted intraperitoneal (i.p.) injection of 80 mg/kg ketamine chlorohydrate solution (Merial, Barcelona, Spain) and 10 mg/kg 2% xylacine chlorhydrate (Bayer, Barcelona, Spain). For surgery, the body temperature of the animals was maintained by placing them on a 37.5 °C heating platform.

All optic nerve crushes were performed on the left eye. Using 2.5 mm Vannas Spring Scissors (Fine Science Tools GmbH, Heidelberg, Germany), the conjunctiva and tenon was opened from the 1 o’clock to the 3 o’clock position. By slightly rotating the eyeball nasally and using fine forceps the optic nerve was carefully exposed. Using self-closing negative action tweezers (Dumoxel Dumont Negative Action Tweezers, Style N7) the optic nerve was crushed for 7 s at a site between 1 and 2 mms behind the globe. During surgery both eyes were moisturized with artificial eye drops, which was continued on the non-surgery eye until the animals woke up again. After surgery ofloxacin eye ointment was applied to the left, surgery eye and all mice received an intraperitoneal injection with Buprenorphine (Buprenorphine; Bedford Laboratories, Bedford, OH, USA). Mice were sacrificed 7 days after the surgery.

Brn3a staining in the cryosections was performed as previously published [17]. To stain RGCs with Brn3a in the cryosections, the sections were re-fixed with 4% PFA for 10 min, washed 3× with PBS for 10 min, then treated with 10 mM citrate buffer and heated in the microwave, and subsequently permeabilized with 1% Triton 4 × 20 min. After washing, the sections were blocked with BGT (0.3% BSA, 0.75% glyine, 0.25% triton X-100) for 1h and then the primary antibody (mouse anti-Brn3a, MAB1585, Millipore, Burlington, MA, USA) was incubated over night at 4 °C. The following day the sections were incubated with the secondary antibody for 1h at RT and mounted in Fluoromount-G (SouthernBiotech, Birmingham, AL, USA) with DAPI 1:1000. Pictures were taken with a Leica SP5 confocal microscope using x63 objective. 4 pictures per retina were taken; Brn3a staining was counted manually in a masked manner using ImageJ cell counter plugin. 

### 4.8. Statistical Analysis

Data are presented as the mean +/− the standard error of the mean of at least three independent experiments performed in triplicate. Differences between treatments were analyzed using a Student’s *t*-test, ANOVA or in the case of non-normally distributed data, a non-parametric Mann–Whitney *U*-test (SPSS 17.0, IBM, Armonk, NY, USA). Statistical significance was set at *p* < 0.05.

## Supplementary Materials

Supplementary materials can be found at https://www.mdpi.com/1422-0067/21/5/1882/s1.

## Abbreviations

AO	Antimycin/Oligomycin
ARPE	Adult Retinal Pigment Epithelial
CCCP	Carbonyl cyanide m-chlorophenyl hydrazone
DAPI	4’,6-Diamidin-2-phenylindol
DFO	Deferoxamine
DFP	Deferiprone
DIC	Differential interference contrast
DIV	Day(s) in vitro
IOP	Intra ocular pressure
MRT	MRT 68921 dihydrochloride
ONC	Optic nerve crush
ONL	Outer nuclear layer
PLL	Poly-L-lysine
PQ	Paraquat
RGC	Retinal ganglion cell
RGCL	Retinal ganglion cell layer
ROS	Reactive oxygen species
STS	Staurosporine
tBOOH	*tert*-Butyl hydroperoxide
