# Supplementary material for: The mito-QC Reporter for Quantitative Mitophagy Assessment in Primary Retinal Ganglion Cells and Experimental Glaucoma Models
Source: Int J Mol Sci. 2020 Mar 10;21(5):1882. doi: 10.3390/ijms21051882 (PMC7084520; doi:10.3390/ijms21051882)
Supplement: Supplementary file 1 [file ijms-21-01882-s001.pdf]

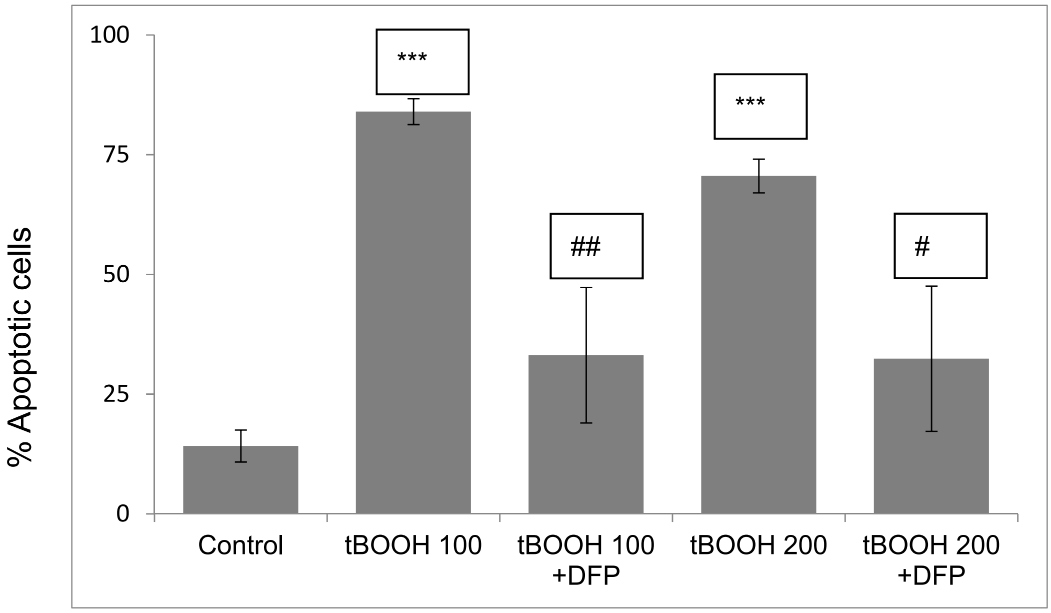

Supplementary Figure 1: ARPE19 *mito*-QC cells were incubated with the indicated doses of tBOOH with or without the presence 0.25 mM DFP for 24h. Cells were fixed and stained for anti-caspase-3. The % of activated caspase-3 apoptotic cells is displayed. *** p<0.001 *vs.* control, # p<0.05 *vs.* treatment, ## p<0.01 *vs.* treatment.

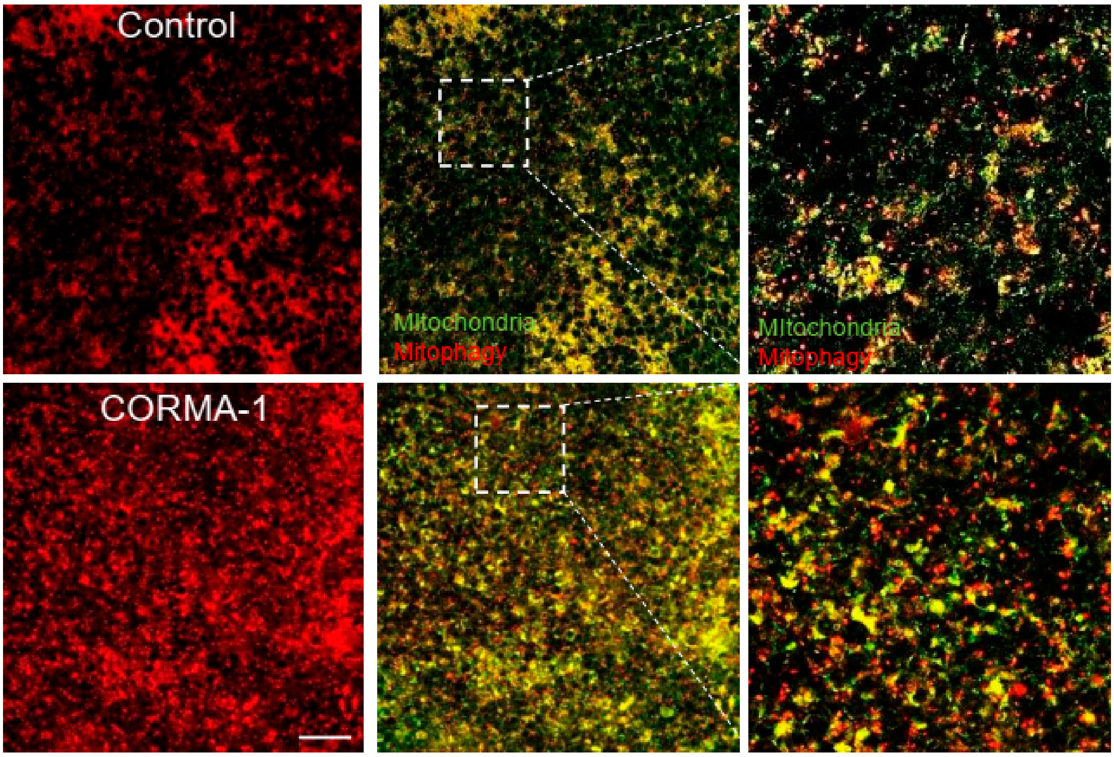

Supplementary Figure 2: Mitophagy of the ONL of retinal *ex vivo* explants cultured with CORM-A1 was assessed. The pictures show exemplary DAPI pictures as well as the red only channel, and an overlay of the red and green channel; highlighting the red puncta in a magnification, scale bar 10 µm.
